# Importance of Hypervariable Region 2 for Stability and Affinity of a Shark Single-Domain Antibody Specific for Ebola Virus Nucleoprotein

**DOI:** 10.1371/journal.pone.0160534

**Published:** 2016-08-05

**Authors:** George P. Anderson, Daniel D. Teichler, Dan Zabetakis, Lisa C. Shriver-Lake, Jinny L. Liu, Stephen G. Lonsdale, Sarah A. Goodchild, Ellen R. Goldman

**Affiliations:** 1 Center for Bio/Molecular Science and Engineering, Naval Research Laboratory, Washington, DC, United States of America; 2 Science and Engineering Apprenticeship Program, Naval Research Laboratory, Washington, DC, United States of America; 3 Defence Science and Technology Laboratory, Porton Down, Salisbury, United Kingdom; National Cancer Institute, UNITED STATES

## Abstract

Single-domain antibodies derived from the unique New Antigen Receptor found in sharks have numerous potential applications, ranging from diagnostic reagents to therapeutics. Shark-derived single-domain antibodies possess the same characteristic ability to refold after heat denaturation found in single-domain antibodies derived from camelid heavy-chain-only antibodies. Recently, two shark derived single-domain antibodies specific for the nucleoprotein of Ebola virus were described. Our evaluation confirmed their high affinity for the nucleoprotein, but found their melting temperatures to be low relative to most single-domain antibodies. Our first approach towards improving their stability was grafting antigen-binding regions (complementarity determining regions) of one of these single-domain antibodies onto a high melting temperature shark single-domain antibody. This resulted in two variants: one that displayed excellent affinity with a low melting temperature, while the other had poor affinity but a higher melting temperature. These new proteins, however, differed in only 3 amino acids within the complementarity determining region 2 sequence. In shark single-domain antibodies, the complementarity determining region 2 is often referred to as hypervariable region 2, as this segment of the antibody domain is truncated compared to the sequence in camelid single-domain antibodies and conventional heavy chain variable domains. To elucidate which of the three amino acids or combinations thereof were responsible for the affinity and stability we made the 6 double and single point mutants that covered the intermediates between these two clones. We found a single amino acid change that achieved a 10°C higher melting temperature while maintaining sub nM affinity. This research gives insights into the impact of the shark sdAb hypervariable 2 region on both stability and affinity.

## Introduction

Both camelids and sharks produce unique heavy chain antibodies that are able to recognize their cognate antigen with excellent affinity and specificity in the absence of a light chain [1,2]. Binding takes place through an unpaired variable heavy domain which can be expressed recombinantly as a single-domain antibody (sdAb) [3,4,5,6]. The single domain architecture of sdAb provides recognition reagents with properties such as good solubility, facile production in *Escherichia coli*, and the ability to refold after heat denaturation. There have been many reports on techniques to improve the properties of camelid-derived sdAb through protein engineering including engineering stabilizing disulfide bonds, introduction of negative charges, addition of biotinylation sequences, humanization, and complementarity determining region (CDR) grafting [7,8,9,10,11,12,13,14]. However to date there are only a handful of reports detailing the engineering of shark-derived sdAb, *i*.*e*.: humanization, affinity maturation, introduction of dimer-forming domains, and linked sdAb [15,16,17,18].

Shark sdAb have been developed towards a number of targets relevant to human health; recently shark sdAb that recognize the nucleoprotein (NP) of the Ebola virus (EBOV; formerly the Zaire ebolavirus) have been described [19]. Although demonstrated in binding assays with live virus [19,20], neither the thermal stability of these sdAb nor their binding kinetics were evaluated. We prepared and evaluated these shark sdAb and found that while they had excellent binding affinity to a truncated recombinant NP construct, they showed melting temperatures of only ~50°C. In this work we focused on improving the stability of one of these shark-derived sdAb.

Previously, we had characterized the stability and refolding ability of a panel of sdAb derived from both spiny and smooth dogfish shark. That work identified several sdAb, such as SP15 that had melting temperatures of 72°C [21]. We chose the SP15 sdAb’s framework for a CDR grafting strategy to improve the stability of the anti-EBOV shark-derived sdAb. CDR grafting techniques have been applied to camelid sdAb and conventional antibody variable domains for improving stability [14,22,23,24]. However, to our knowledge CDR grafting had not been reported for shark-derived sdAb for the purposes of increasing thermal stability.

In this report we utilized CDR grafting combined with site directed mutagenesis to increase the melting temperature (stability) of a shark derived anti-NP sdAb. Two derivatives of the shark sdAb were constructed: One where CDRs 1 and 3 along with HV2 (the designation given to the CDR2 region of shark sdAb) were grafted onto the framework of SP15, and another in which only CDRs 1 and 3 were grafted. Interestingly, we found that the HV2 region, which is located somewhat remote from antigen-binding CDR1 and CDR3 regions, had significant impact on both affinity and stability. This work elucidates the importance of the HV2 region on both folding stability and antigen binding.

## Materials and Methods

### Reagents

Our previously reported shark sdAb were utilized to provide a framework for CDR grafting [21]. DSTL096 and DSTL097 protein samples were prepared as previously described [19]. All cloning enzymes were from New England Biolabs unless otherwise indicated. Oligonucleotides, synthetic genes, and DNA sequencing services were purchased from Eurofins Genomics. Nucleoprotein (NP) was from Sino Biological Inc. Viral Protein 40 (VP40) and glycoprotein (GP) were from IBT Bioservices.

The deduced amino acid sequence for the sdAb-domain of DSTL096 was previously published [19]. The shark sdAb shark096 as well as the graft constructs (SP15-096-123, SP15-096-13) and point mutants (E to W, S to R, K to I, EK to WI, SK to RI, ES to WR) were synthesized by Eurofins Genomics to include unique NcoI and NotI restriction endonuclease sites flanking the coding sequence for the sdAb to enable cloning into the pET22b(+) periplasmic expression vector.

### Protein production

Shark sdAb were purified essentially the same as previously described [21,25]. Protein expression was achieved utilizing the *E*. *coli* strain BL21(DE3) that had been transformed with both the pET22b(+)-based sdAb expression plasmids and the pHELP1 plasmid, expressing the *E*. *coli* Skp chaperone gene [26]. Bacteria were grown from freshly transformed colonies in 50 mL terrific broth containing Ampicillin (100 μg/mL) and Chloramphenicol (30 μg/mL). All growth in liquid media for protein expression was at 25°C. Fifty mL overnight cultures were added to 450 mL terrific broth (containing both antibiotics) and grown 3 hours. Arabinose (0.8 mg/mL final concentration) was added to the cultures which were grown for one half hour before being induced with IPTG (0.25 mM final concentration).

After induction, cultures were grown an additional 2 to 3 hours and then the cells were pelleted by centrifugation and subjected to an osmotic shock protocol and protein purified by immobilized metal affinity chromatography followed by size exclusion chromatography. Cell pellets were suspended in 14 ml ice-cold sucrose-tris (750 mM sucrose, 100 mM Tris pH 7.5), and then 28 mL of 1 mM ethylenediaminetetraaceticacid (EDTA; pH 8) was added drop-wise to each sample. The cells were swirled gently for 15 min on ice, and then 1 mL of 500 mM MgCl_2_ was added and the samples incubated on ice a further 10 minutes before pelleting. Supernatants were poured into 50-mL conical tubes. Five mL of 10 x IMAC buffer (0.2M Na_2_HPO_4_, 4 M NaCl, 0.2 M imidazole, pH 7.5) and 0.5 ml of Ni Sepharose (GE Healthcare) that had been washed with 1x IMAC buffer, were added to the supernatant and the sample tumbled on a rotisserie at 4°C on overnight. The next morning, the resin was washed two times in batch with 30–40 mL of 1 x IMAC buffer, then the resin was packed into a small column and bound sdAb eluted with 1 x IMAC buffer containing 500 mM imidazole. Further purification was achieved by size exclusion chromatography using a Superdex 75 10/300 GL column and a Bio-Rad Duo-Flow System.

Yield of the sdAb was determined by UV spectroscopy, measuring absorbance at 280 nm using a Nanodrop (ThermoFisher). Yields were determined from at least two independently produced batches of protein that were purified on different days.

Selected sdAb were also produced as above except that the pTUM4 plasmid [27] was used instead of pHELP1. In this case cultures were only induced with IPTG. Likewise, proteins were also produced by growing BL21(DE3) transformed only with the pET22b(+)-based sdAb expression plasmids. When only the pET22b(+) expression plasmid was utilized, ampicillin was the only antibiotic added and cultures were only induced with IPTG.

### Surface plasmon resonance

Surface plasmon resonance (SPR) affinity and kinetics measurements were performed using the ProteOn XPR36 (Bio-Rad). Lanes of a general layer compact (GLC) chip were individually coated with recombinantly produced EBOV proteins NP, GP and VP 40. Immobilization of the EBOV proteins was performed using proteins diluted in 10 mM acetate buffer pH 5.0 and attached to the chip following the standard 1-ethyl-3-(3-dimethylaminopropyl)carbodiimide hydrochloride (EDC) coupling chemistry available from the manufacturer. Binding kinetics of each sdAb was tested at 25°C by flowing six concentrations varying from 300 to 0 nM at 100 μL/min for 90 s over the antigen coated chip and then monitoring dissociation for 600 s. This generates binding data for each of the antigens immobilized on the chip. Following each run, the chip was regenerated by flowing 0.085% phosphoric acid (~pH 3.0) across the surface for 18 s. Data analysis was performed with ProteOn Manager 2.1 software, corrected by subtraction of the zero antibody concentration column as well as interspot correction. The standard error on the fits was less than 10%. Binding constants were determined using the Langmuir model built into the analysis software. Measurements for DSTL096, shark096 and their derivatives were determined from at least two independently produced batches of protein that were purified at separate times.

### Circular dichroism

Refolding and melting temperatures were assessed by circular dichroism (CD) using a Jasco J-815 CD spectrometer as described previously [21]. Proteins were prepared in deionized water at a final concentration of about 12 μg/mL. CD was monitored at a single wavelength between 200 and 208 nm as samples were heated from 25°C to 95°C at a rate of 2.5°C/min. The melting temperature is taken to be the inflection point of the S-shaped curve of ellipticity versus temperature. A cooling cycle was carried out at the same rate and the CD monitored to determine the ability to regain secondary structure after heat denaturation. Melting temperatures determined from runs using different protein preparations were within 1°C and were thus considered identical.

### Fluorescence-based melting assay

The melting temperature of each sdAb was measured by a fluorescent dye-based assay as outlined previously [21]. This technique relies on the fluorescence enhancement of Sypro Orange (Sigma), as it interacts with the hydrophobic amino acids on a protein, which become accessible upon thermal unfolding. Each sdAb was diluted to a concentration of 500 μg/mL in a final volume of 20 μL PBS. The Sypro orange dye was added to each sample at a dilution of 1:1000. Samples were measured in triplicate using a StepOne Real-Time PCR machine (Applied Biosystems). The heating program was run in continuous mode from 25°C– 99°C at a heating rate of 1% (~2°C per minute). Data was recorded using the ROX filter and the melting point was determined to be the peak of the first derivative of the fluorescence intensity. All three replicates gave essentially identical values for the melting temperature. Additionally, melting temperatures for DSTL096, shark096 and their derivatives were determined from at least two independently produced batches of protein.

## Results

### Characterization of the original shark anti-EBOV NP sdAb

We determined the binding affinity and melting temperature of two previously described anti-EBOV sdAb-fusion constructs, DSTL096 and DSTL097. These antibodies are based on sdAb which were selected from a phage display library derived from a shark immunized with inactivated EBOV and found to bind the NP [19]. Both of DSTL096 and DSTL097 were determined to have sub-nM affinities for a construct consisting of the C-terminal portion of NP. Circular dichroism showed their melting temperature to be 53 and 55°C respectively and both refolded at least 65% after heat denaturation (Table 1).

**Table 1:** Characterization of DSTL096, DSTL097, shark096 and original graft constructs.

Clone	Tm, Dye melt ^a^, °C	Tm CD ^a^, °C (% refold)	K_D_ ^b^ (standard deviation), M
DSTL096	49	53 (68)	3.9 E-10 (1.5 E-10)
DSTL097	50	55 (65)	7.0 E-10 (3.5 E-10)
Shark096	50	52 (74)	3.4 E-10 (3.3 E-10)
SP15-096-123	53	53 (51)	3.7 E-10 (1.1 E-10)
SP15-096-13	68	68 (73)	2.7 E-08 (6.3 E-09)

^a^ Values for the melting temperature (Tm) determined by the dye melt and circular dichroism (CD) methods were within 1°C on independent measurements, including those utilizing proteins prepared at different times.

^b^ Dissociation constants (K_D_) were determined by surface plasmon resonance (SPR).

**Fig 1:**
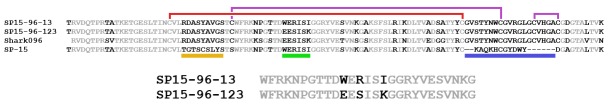
Protein sequences of parental and graft variants. Protein sequence of DSTL096, the SP-15 stable sdAb framework donor, and the two graft mutants are shown in the top sequence comparison. Conserved sequences are shown in gray, and differences in black. The CDR regions are indicated by colored bars (orange for CDR1, green for HV2, and blue for CDR3). The region designated as HV4 [15,40] is indicated by a gray bar, and was considered as framework for this work. The natural canonical and non-canonical disulfide bonds are indicated by lines joining the cysteines, with the canonical in red and non-canonical in purple. The bottom panel shows a close up of the area containing the three positions that differ between SP15-096-13 and SP15-096-123. Point mutations were constructed to produce the six intermediate proteins.

**Fig 2:**
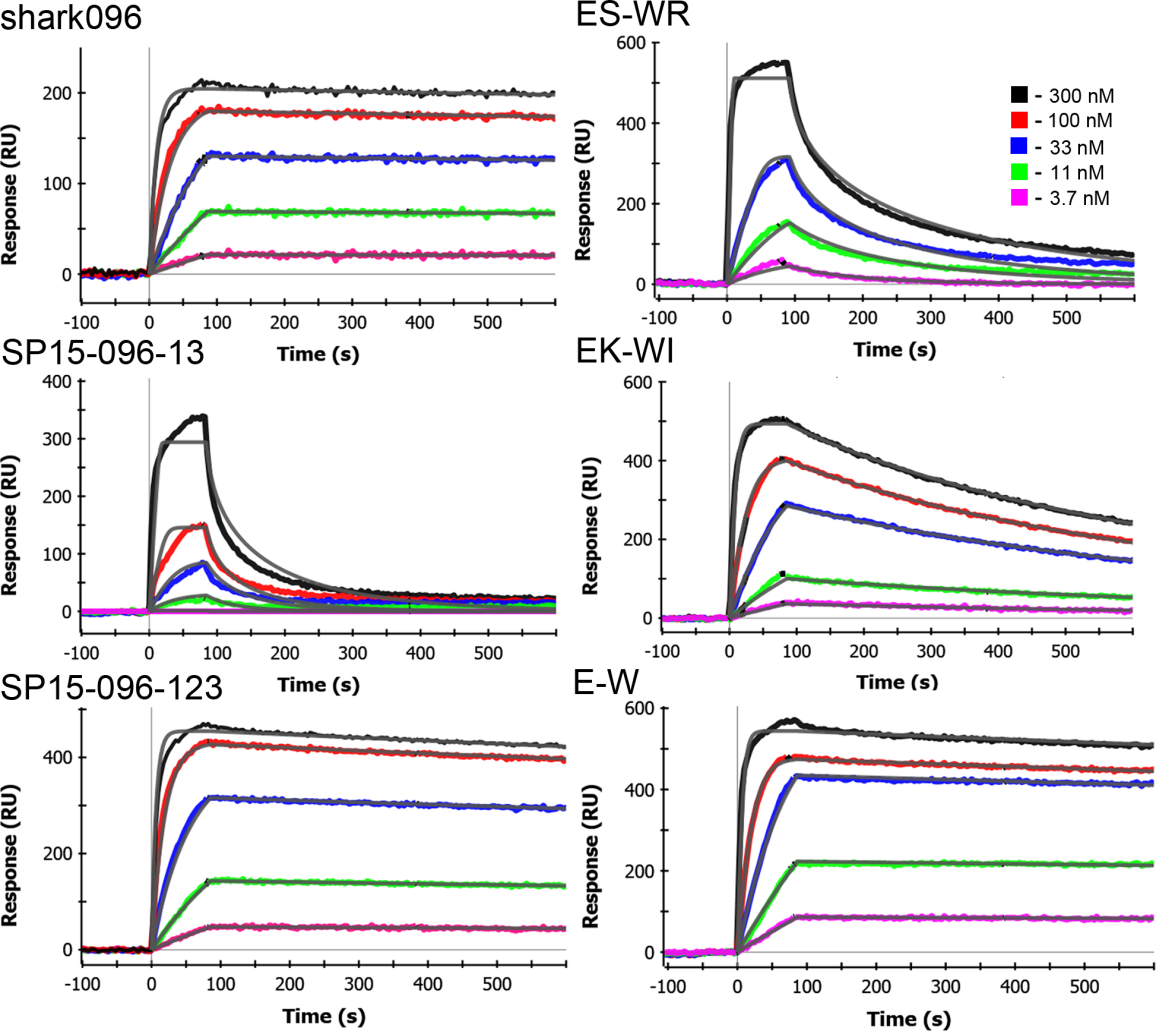
Representative surface plasmon resonance data. SPR data showing binding to the immobilized recombinantly expressed NP construct. The parental sdAb, Shark096 as well as the two starting CDR graft variants are shown on the left while the right shows three of the point mutants including the EK to WI and E to W mutant that showed the highest melting temperature and affinity combinations. The legend shows the concentration of sdAb in nM; the calculated fit is shown as a gray line. Error on the fits was within 10%. This is representative data chosen from at least three separate kinetics measurements for each of these sdAb variants.

The DSTL096 and DSTL097 constructs are composed of the sdAb expressed as a fusion with a murine kappa light chain constant domain. An unfused construct containing only the sdAb portion of DSTL096 was prepared using its published protein sequence (Fig 1). The gene was synthesized and cloned into the pET22b(+) expression vector to produce the construct shark096. Other researchers had validated the ability of both the unfused shark anti-EBOV NP sdAb to bind live virus [20]. Similarly, our experiments confirmed that shark096 has essentially identical affinity and melting characteristics to the fusion construct with a K_D_ = ~ 0.3 nM (Fig 2) and melting temperature of 52°C (Table 1). Using SPR we also verified the specificity of shark096, showing that it had no cross reactivity to other recombinant EBOV proteins (VP40, GP; data not shown).

### Protein production

The shark096 sdAb, and variants thereof, were produced using the same protocol that generates high yields from llama-derived sdAb utilizing the pET22b(+) expression vector [11,28]. We have found that shark sdAb, in general, do not produce as well as the llama variants. Our yield for shark096 was consistently between 1 and 2.5 mg purified protein per L of *E*. *coli*. Yields of SP-15 were previously determined to be ~ 4 mg/L, one of the highest we had achieved from bacterial expression of a shark sdAb. Yields for the grafts of the shark096 CDRs onto the SP-15 framework, however, were only ~ 1 mg/L, while those for the point mutant versions described below ranged from ~0.5 to 2 mg/L

It has been found that addition of a non-canonical disulfide bond to the llama sdAb can decrease protein production [8,28,29]. The shark096 construct has two natural non-canonical disulfide bonds, and it was hypothesized that the additional disulfide bonds might at least partially explain the poor production. When the four cysteines involved in shark096’s additional disulfide bonds were mutated, the protein production increased several fold, however no binding to target was observed. We tested if the yield of shark096 could be increased by co-expression of the sdAb with additional DsbA and DsbC, proteins involved in the periplasmic formation of disulfide bonds, by utilizing the pTUM4 plasmid to correct any improperly formed disulfide bonds. While pTUM4 has proven useful for production of several proteins with multiple disulfide bonds [27], in this case the improvement was at best two-fold over the yield in the absence of any chaperone and equivalent to production when using the pHELP1 plasmid [26]. Potentially moving to an alternate production system such as yeast could prove a successful strategy for scaling up production of these proteins [30,31].

### Evaluation of shark096 CDR grafts

A CDR grafting strategy was employed in an effort to increase the melting temperature of shark096. Using the framework of a sdAb derived from spiny dogfish shark which had demonstrated good thermal stability [21], we had CDR grafts synthesized. The starting sdAb, SP-15 had a melting temperature of 72°C as assessed by CD, refolded well, and produced at a yield of ~ 4 mg/L.

CDR graft constructs were synthesized which included all 3 CDR regions of shark096 or only CDR regions 1 and 3. This resulted in 2 initial grafts named for the framework and the CDRs that were grafted. Clones are named by listing the framework (SP15 for SP-15) followed by “096” and then the CDRs from shark096 that were grafted (123 or 13) and are as follows: SP15-096-123 and SP15-096-13. Sequences were aligned using MultAlin [32] and are shown in Fig 1. First, we used the dye-melt assay to determine melting temperatures of the clones. The SP15-096-123 variant had a low melting temperature (53°C) similar to the parental shark096. SP15-096-13, the version lacking the HV2 (CDR2), had an elevated melting temperature of 68°C. Using SPR, we examined the ability of the sdAb to bind the recombinatly expressed NP construct. The sdAb construct which lacked the HV2 graft showed poor binding (tens of nM), while the graft which included HV2 showed sub-nM binding within a factor of 2 of the K_D_ determined for DSTL096. Table 1 shows characterization of grafts, representative SPR data is shown in Fig 2.

### Evaluation of single and double point mutants

There were only three amino acid differences in the HV2 region of SP15-096-13 which had higher stability and SP15-096-123 which had better affinity (Fig 1). We prepared the six single and double point mutants to examine all possible sequence variants between these two sdAb, in an attempt to derive a variant that demonstrated improved stability while retaining the high affinity of parental shark096. The six point mutants are named to show their deviation from the SP15-096-123 base sequence and are named: E to W, S to R, K to I, EK to WI, SK to RI, and ES to WR.

The melting temperatures and affinity (K_D_) of the point mutants are shown in Table 2; representative SPR data is shown in Fig 2. Each of the changes led to increases in melting temperature. The increases for the double mutants appear to be approximately the sum of their cognate single mutant increases. The highest melting temperature increase was seen in the EK to WI mutant, with a melting temperature of 66°C, close to that measured in the SP15-096-13 graft. The E to W mutation led to a melting temperature of 61°C, the highest increase seen among the three single mutations. Affinity measurements showed that only the E to W mutant possessed sub nM affinity, similar to the parental shark096. The EK to WI mutant, which had the highest gain in melting temperature displayed low nM affinity.

**Table 2:** Characterization of point mutants.

Clone	Tm, Dye melt ^a^, °C	Tm CD ^a^, °C (% refold)	K_D_ ^b^ (standard deviation), M
E to W	61	63 (78)	3.4 E-10 (1.8 E-10)
S to R	53	-	2.3 E-09 (6.7 E-10)
K to I	58	-	2.2 E-09 (5.8 E-10)
EK to WI	66	66 (75)	3.6 E-09 (1.0 E-09)
ES to WR	62	-	1.2 E-08 (9.2 E-09)
SK to RI	60	-	2.2 E-08 (3.6 E-09)

^a^ Values for the melting temperature (Tm) determined by the dye melt and circular dichroism (CD) methods were within 1°C on independent measurements, including those utilizing proteins prepared at different times.

^b^ Dissociation constants (K_D_) were determined by surface plasmon resonance (SPR).

### Discussion

Camelid and shark-derived sdAb often have the ability to refold after heat denaturation, even when their melting temperatures are relatively low. However variables such as time at the elevated temperature and the concentration of the sdAb contribute to their ability to regain an active secondary structure versus forming aggregates. We have an ongoing interest in the stabilization of camelid-derived sdAb with the end goal of providing robust immunoreagents that could maintain functionality for extended periods of time in austere environments. Towards this goal we have explored several strategies to increase their melting temperature [9,11,22]. In this work, we extended our efforts to the stabilization of a shark-derived sdAb.

Our starting point for this work was the shark-derived sdAb, DSTL096, derived from a shark immunized with inactivated EBOV and specific for EBOV NP [19]. The DSTL096 construct consists of a fusion of the shark sdAb with a murine kappa light chain constant domain. Previously, DSTL096 was shown to bind live virus and found to retain about 80% of its binding ability after heating to 70°C for 3 hours [19], however neither the sdAb’s affinity for NP nor melting temperature was measured. In this work we found that DSTL096 had sub-nM affinity for a recombinantly produced NP c-terminal fragment, however according to CD, it melted at only 53°C. Likely, the retention of activity after heating reported in the previous work was due to refolding, as CD showed the construct refolded (~68%) on cooling after heat denaturation.

For our stabilization studies, we generated an expression construct to prepare the sdAb anew, without a fusion partner other than a polyhistidine tag (shark096). Upon testing, it was found to behave similar to the DSTL096 construct with excellent affinity (K_D_ = ~ 0.3 nM), and a melting temperature of 52°C (measured by CD). We were surprised at the low melting temperature of this shark sdAb given that in addition to the canonical disulfide found in the variable regions of antibodies, shark096 has four additional cysteine residues that presumably form two additional non-canonical disulfide bonds. The presence of these additional non-canonical disulfide bonds is characteristic of the Type 1 class of shark sdAb [3]. In previous studies utilizing camelid sdAb, the addition of non-canonical disulfide bonds was often correlated with having higher melting temperatures [8,9]. This increase was 20°C in the case of one llama derived sdAb [33].

We started with a CDR grafting strategy in an effort to increase the melting temperature of shark096 without compromising its excellent affinity for EBOV NP. Mutagenesis to change the four cysteines involved in shark096’s additional disulfide bonds led to a protein that no longer recognized antigen, indicating that in this case the non-canonical disulfides act to position the CDR3 rather than to stabilize the structure. Therefore, we preserved the cysteine residues in the shark096 framework to maintain antigen recognition.

Previously, we had performed a survey of sdAb derived from spiny dogfish shark and smooth dogfish shark examining biophysical characteristics including protein production, melting temperature, and refolding after thermal denaturation [21]. In that work, we randomly selected sdAb from naïve libraries, so no affinity measurements were obtained, as the cognate antigen of the sdAb being evaluated was unknown. However, several sdAb were identified with melting temperatures greater than 70°C, the ability to refold after heat denaturation, and an acceptable level of protein production. For the current effort we selected a spiny dogfish shark framework (SP-15) as suitable frameworks for CDR grafting.

Although the variable domains from shark heavy-chain-only antibodies have β-sandwich fold characteristic of the Ig superfamily, they consist of 8 β strands in contrast to the 10 found in variable regions from conventional antibodies and camelid sdAb [2,34,35,36]. The CDR2 region is truncated in shark derived sdAb [37] and the short strand in place of CDR2 is also referred to as hyper variable region 2 (HV2) [36]. Most of the variability of shark sdAb is contained in the CDR3 regions; in crystal structures of shark sdAb complexed with antigen the CDR2 (HV2) region does not play a direct role in binding [15,36]. However, a randomized library of the HV2 region showed that it is capable of mediating antigen binding [38]. Hence we chose to make two sets of grafts: one in which all three CDRs were grafted on to the frameworks, and a second in which only CDR 1 and 3 were grafted.

The most successful graft in terms of thermal stabilization was SP15-096-13. This construct, which retained the HV2 of SP15, increased the melting temperature by 17°C to 68°C but had poor affinity. On the other hand version SP15-096-123, which had all 3 CDRs of shark096, had virtually unchanged stability and affinity when compared to shark096, Table 1.

**Fig 3:**
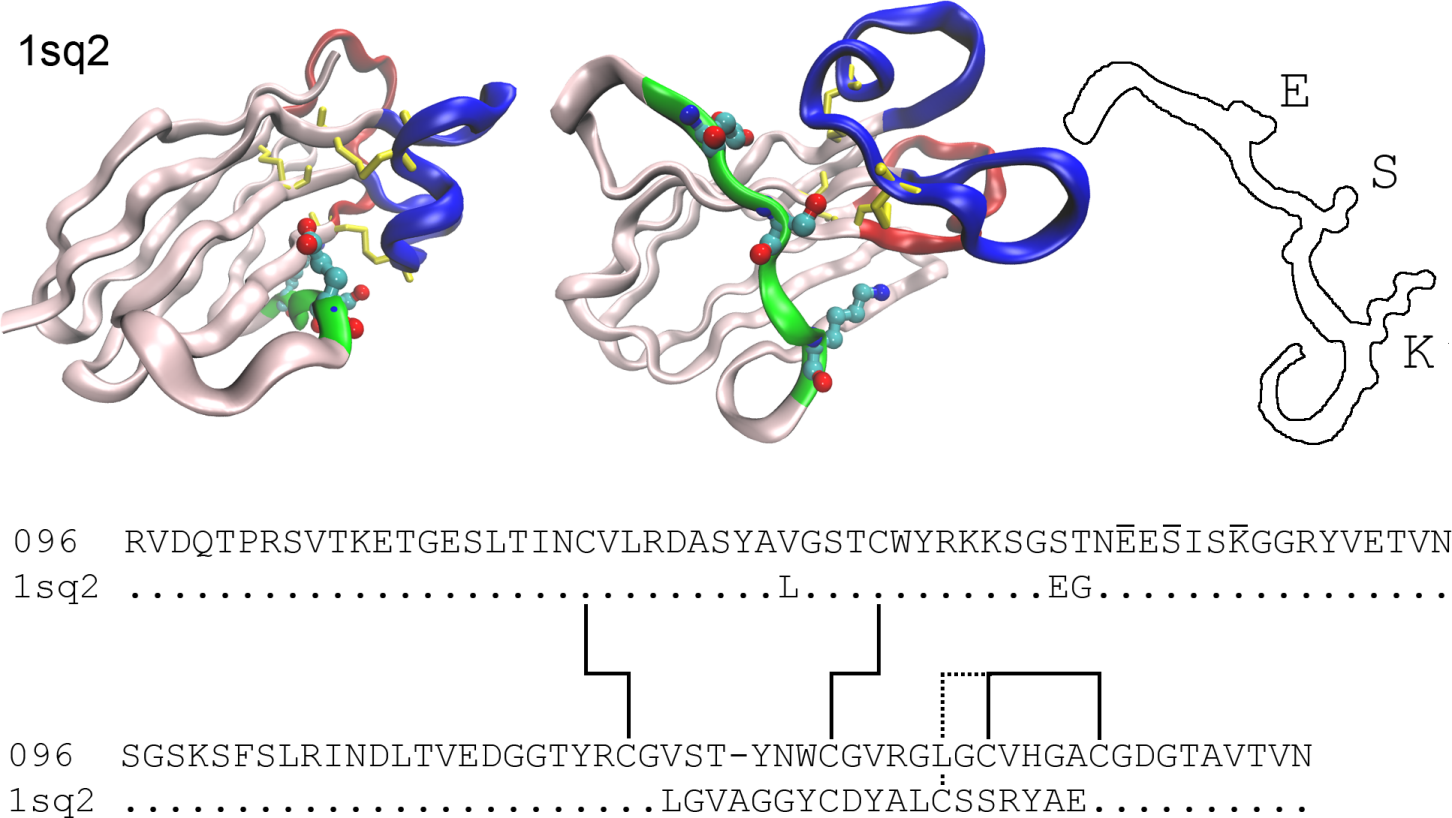
Schematic of the shark096 sdAb structure. Two views of a nurse shark sdAb structure [36] (PDB #1sq2) with high sequence homology to shark096. The CDR regions are color coded red, green, blue for CDRs 1 2 and 3 respectively and disulfide bonds are in yellow. In ball and stick are shown the position of the three amino acids that differed between the two graft structures (SP15-096-123 and SP15-096-13). The third panel shows the specific residues for clarity. The sequence of shark096 is shown and that for 1sq2 is shown as a difference plot. Dots indicate identical residues. The disulfide bonds are indicated by lines and the three mutated residues are identified by overscores. Although the CDR2 region is truncated and presumably has no contact with antigen, it was found critical for both stability and affinity.

One strategy towards achieving increased stability would be to fully randomize the whole HV2 region. However, as SP15-096-13 had a melting temperature of 68°C and sequence comparison showed only three amino acid differences with SP15-096-123 which retained a high affinity (Fig 1), we elected to prepare a series of point mutations in an effort to produce a superior construct. Three single and three double point mutations were constructed which represent all combinations of the differences between the two proteins. Fig 3 shows a structure of a shark sdAb with high homology to shark096 [36] highlighting the positions of the three HV2 residues at which we targeted mutations. Testing these six mutants we found that the single point E to W mutant possessed sub nM affinity (0.34 nM) and a melting temperature ~10°C better than the parental shark096, Table 2. The most stable of the point mutants (EK to WI) had a melting temperature about 15°C better than shark096, and 3.6 nM affinity, however, it may still be sufficient for use in detection assays in austere locations where higher stability is essential for retention of binding activity.

Fig 3 shows the crystal structure of Protein Data Bank entry 1sq2 which is a nurse shark IgNAR in complex with lysozyme. This antibody has a high sequence homology with shark096 except for CDR3. In particular the disulfide bonds are located in essentially similar contexts. We therefore take this structure as a reasonable representation of shark096, thereby giving us a reasonable picture of where the three differing amino acids are located. Each of the three residues is located on an inward-facing side of the protein strand, and in close association with CDR3. While sequence differences prohibit a detailed interpretation of these interactions, this structure supports the hypothesis that these residues can modulate the binding of antigen via CDR3. Other researchers have noted the importance of the HV2 (CDR2) region for interaction with antigen. In particular, in the case of a human serum albumin (HSA) binding shark sdAb, it was noted that the HV2 forms a network of hydrogen bonds that serve to orient other amino acid residues for interactions with HSA [15]. The authors postulate that disrupting the hydrogen binding network would adversely affect antigen binding through impacting the position of amino acids that directly interact with antigen.

Shark derived sdAb offer an important alternative to both conventional antibodies and camelid sdAb for therapeutic, detection, and biotechnology applications [6,34,39]. Through CDR grafting, coupled with site directed mutagenesis, we were successful in finding a variant of shark096 that has an increased melting temperature while maintaining high affinity. The mutant E to W offered a ~10°C improvement in melting temperature while maintaining an affinity for NP approximately the same as shark096. A future focus will be on improving the protein production of this clone.

To our knowledge this is the first demonstration of molecular engineering to increase the thermal stability of a shark sdAb. In addition, this work highlights the potential importance of the HV2 region for both affinity and stability of shark-derived sdAb.
